# The evaluation of the aortic annulus displacement during cardiac cycle using magnetic resonance imaging

**DOI:** 10.1186/s12872-018-0891-4

**Published:** 2018-07-31

**Authors:** Tomasz Plonek, Mikolaj Berezowski, Jacek Kurcz, Przemyslaw Podgorski, Marek Sąsiadek, Bartosz Rylski, Andrzej Mysiak, Marek Jasinski

**Affiliations:** 1 Department of Cardiac Surgery, Wroclaw Medical University, Borowska 213, 50-556, Wroclaw, Poland; 2 Department of Cardio-Thoracic Surgery, Onze Lieve Vrouwe Gasthuis, Amsterdam, the Netherlands; 3 Department of General and Interventional Radiology and Neuroradiology, Wroclaw Medical University, Wroclaw, Poland; 4 Department of Cardio-vascular Surgery, Heart Centre Freiburg University, Faculty of Medicine, University of Freiburg, Freiburg, Germany; 5 Department of Cardiology, Wroclaw Medical University, Wroclaw, Poland

**Keywords:** Aorta, Dissection, Aneurysm, Cardiovascular magnetic resonance

## Abstract

**Background:**

The stress in the ascending aorta results from many biomechanical factors including the geometry of the vessel and its maximum dimensions, arterial blood pressure and longitudinal systolic stretching due to heart motion. The stretching of the ascending aorta resulting from the longitudinal displacement of the aortic annulus during the heart cycle has not been examined in the general population so far. The aim of the study is to evaluate this parameter using cardiovascular magnetic resonance (CMR) imaging in the general population in all age groups.

**Methods:**

The cardiac magnetic resonance images of 73 patients were evaluated. The maximum distance to which the ventriculo-aortic junction was pulled by the contracting heart (LDAA – longitudinal displacement of the aortic annulus) was measured in the cine coronal sequences. Moreover, the maximum dimensions of the aortic root and the ascending aorta were assessed.

**Results:**

The LDAA value was on average 11.6 ± 2.9 mm (range: 3-19 mm; 95% CI: 10.9–12.3 mm) and did not differ between males and females (11.8 ± 2.9 mm vs. 11.2 ± 2.9 mm, *p* = .408). The diameter of the ascending aorta was 32 ± 6.3 mm (range: 20-57 mm). The maximal dimension of the aortic root was 35 ± 5.1 mm (range: 18-42 mm). There was a statistically significant negative correlation between the LDAA and the age of patients (*r* = −.38, *p* = .001). There was no significant correlation between the LDAA and aortic root dimension (*r* = .1, *p* = .409) and between the LDAA and diameter of the ascending aorta (*r* = .16, *p* = .170).

**Conclusions:**

Human aortic root and ascending aorta are significantly stretched during systole and the distance to which the aorta is stretched decreases with age. The measurement of the longitudinal displacement of the aortic annulus using the CMR is feasible and reproducible.

## Background

There are several biomechanical factors that influence stress in the wall of the ascending aorta and the risk of aortic dissection. To date, the most common radiographic parameter used to predict this risk is maximum aortic diameter [1–3]. However, recent studies suggest that the aorta usually dissects when its diameter is much lower than the threshold regarded as an indication for surgery [4, 5]. Therefore, new objective parameters to assess the risk of dissection are necessary. Some of them, including the presence of arterial hypertension and genetic predispositions are already taken into account when assessing this risk [2, 3]. Some, new anatomic and radiographic parameters like aortic elongation are being under investigation [6, 7].

The aorta is subjected to circumferential stress since the vessel increases its diameter due to the rise in blood pressure [8, 9]. Moreover, the aortic root and ascending aorta are in close proximity to the heart which pulls and stretches the vessel during systole. This phenomenon results in longitudinal stress [10–12].

The longitudinal displacement of the aortic annulus (LDAA) has not been well examined so far and there are no studies assessing the LDAA with the use of the cardiovascular magnetic resonance (CMR) in the general population [10, 13]. There is only one study that assessed the dynamics of the aorta including the longitudinal strain but included only the elderly population (70 years or older) [14].

This is the first study that uses CMR to evaluate the longitudinal displacement of the aortic annulus in the patients from all age groups. The aim of the study is to estimate the LDAA using magnetic resonance imaging in the general population and assess how it changes with the patients’ age, height and weight.

## Methods

### Patients

This was a retrospective study of all patients who had been referred for cardiovascular magnetic resonance (CMR) between 2015 and 2016 to the Department of General and Interventional Radiology and Neuroradiology at Wroclaw Medical University in Poland. In total, the cardiovascular magnetic resonance images of 89 patients were included for initial assessment. The exclusion criteria were poor quality of the images in the cine coronal view, which resulted mainly from the respiratory movement of the patient (6 patients) or arrhythmia (5 patients). Finally, 73 patients were included in the analysis.

### Cardiovascular magnetic resonance imaging

The CMR examinations were carried out on 1.5 T Signa HDxt (General Electric Healthcare, WI, USA) with an eight phased-array coil. All measurements were performed in the ECG-gated coronal cine Fast Imaging Employing Steady-state Acquisition (FIESTA) sequences. The serial images with a nominal resolution of 1.2 × 1.2 mm and 6 mm section thickness were obtained. The images covered a 256 × 256 mm field of view.

### Measurements

**Fig. 1:**
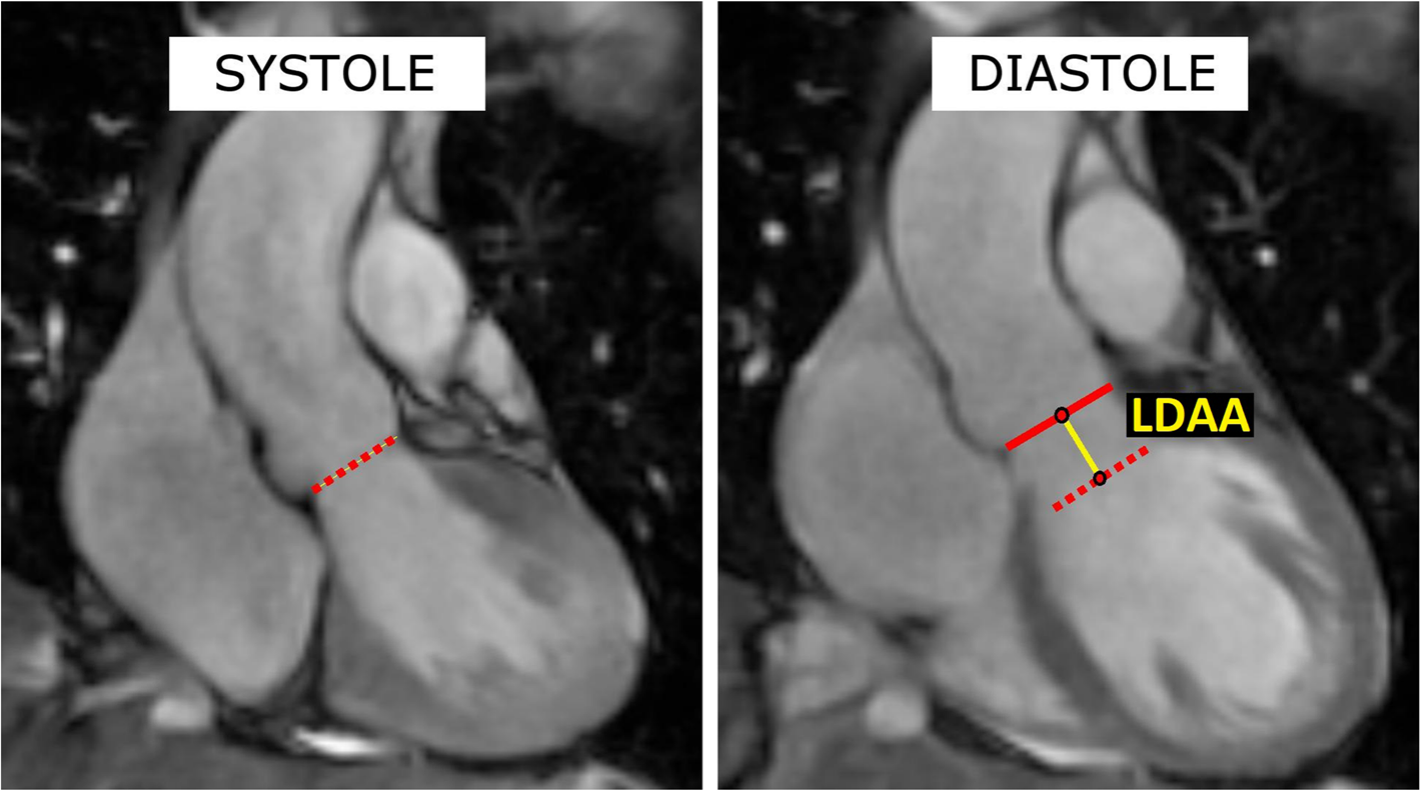
The measurements taken from the coronal cine-MRI sequences. Left (systole): red dotted line - the position of the ventriculo-aortic junction (VAJ) in systole. Right (diastole): red dotted line - the position of the ventriculo-aortic junction (VAJ) in systole, red line - position of the ventriculo-aortic junction (VAJ) in diastole; yellow line – the longitudinal displacement of the aortic annulus (LDAA)- the line links the mid-points of the VAJ in systole and diastole

The CMR images were analyzed in the coronal cine-MRI view. The maximum distance to which the ventriculo-aortic junction (VAJ) was pulled by the contracting heart was measured (LDAA – longitudinal displacement of the aortic annulus). First, the position of the VAJ was established in the maximum systole and diastole, then the distance between the mid-points of the VAJ in systole and diastole was measured. Moreover, the maximum dimensions of the aortic root and the ascending aorta were assessed. The methodology of the measurements is presented in Fig. 1.

### Inter- and intraobserver variability

All examinations were evaluated by two observers. For inter-observer variability, the same examinations were analyzed by a second observer blinded to all the measurements performed by the first observer. For intraobserver variability of longitudinal systolic stretching of the aorta, all measurements were retaken by each observer 1 week after the initial assessment.

### Statistical analysis

The normality of distribution was assessed using the Shapiro-Wilk test. Based on the distribution, data are presented as mean ± standard deviation or median and range for continuous data. The categorical data are presented as frequencies. The correlations were assessed using the Pearson correlation coefficient. The means were compared using either the student’s t-test or the Mann-Whitney U test depending on the normality of the distribution. The inter- and intraobserver variabilities were assessed using the interclass correlation coefficient. The analyses were carried out using Dell Statistica 13 software (Dell, USA).

## Results

### Longitudinal stretching of the aorta

The mean age of the patients was 45.2 ± 17.3 years and 68% (50 patients) were males. The mean height of the patients was 171.3 ± 14.4 cm and mean weight was 77.2 ± 10.8 kg. The mean BMI was 26.1 ± 3.8. There were 11 patients diagnosed with arterial hypertension and one patient had diabetes mellitus. There were no patients with poor ejection fraction of the left ventricle (< 30%). The diameter of the ascending aorta was 32.0 ± 6.3 mm (range: 20-57 mm) and was comparable between the genders (males: 32.6 ± 6.9 mm vs. females: 30.7 ± 4.8 mm, *p* = .256). The maximum dimension of the aortic root was 35.0 ± 5.1 mm (range: 18-42 mm) and was bigger in males than females (36.4 ± 5.2 mm vs. 31.2 ± 3.4 mm, *p* < .001).

**Fig. 2:**
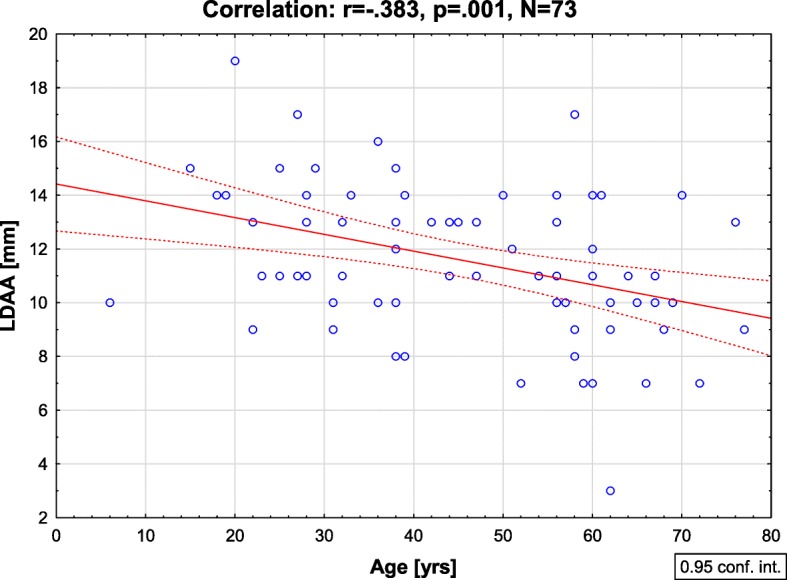
The correlation between the longitudinal displacement of the aortic annulus (LDAA) and the age of the patients

**Fig. 3:**
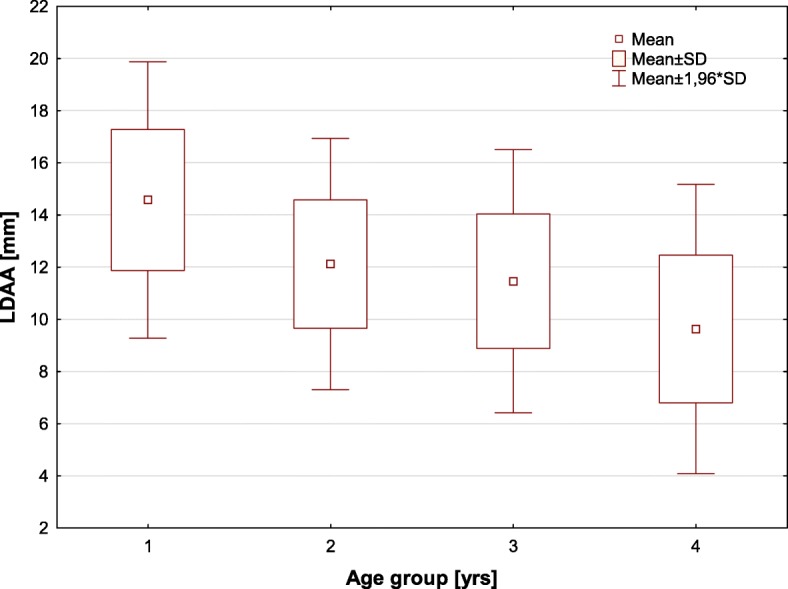
The longitudinal displacement of the aortic annulus (LDAA) in various age groups

The longitudinal displacement of the aortic annulus (LDAA) was on average 11.6 ± 2.9 mm (range: 3-19 mm; 95% CI: 10.9–12.3 mm) and did not differ between males and females (11.8 ± 2.9 mm vs. 11.2 ± 2.9 mm, *p* = .408). The aortic arch (junction between the innominate artery and the left carotid artery) was displaced on average by 2.9 ± 0.4 mm (range: 0-6 mm; 95% CI: 2.1–3.7 mm). There was a tendency towards lower LDAA values in older patients and a statistically significant negative correlation between the LDAA and the age of patients (*r* = −.38, *p* = .001) was observed (Fig. 2). The values of the LDAA in the age groups were as follows (0-20 yrs.: 14.6 ± 2.7 mm; 21-40 yrs.: 12.1 ± 2.5 mm; 41-60 yrs.: 11.5 ± 2.6 mm; 61-80 yrs.: 9.6 ± 2.8 mm; *p* = .002) and are shown on Fig. 3. There was no statistically significant correlation between the LDAA and the patient’s height (*r* = .112, *p* = .092), weight (*r* = .096, *p* = .771) and BMI (*r* = .084, *p* = .501).

**Fig. 4:**
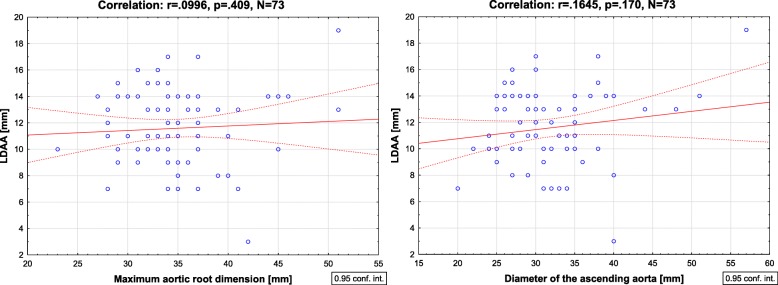
The correlation between the longitudinal displacement of the aortic annulus (LDAA) and the maximum aortic root dimension (left) and the maximum diameter of the ascending aorta (right)

The longitudinal displacement of the aortic annulus was not affected by the maximum dimension of the aorta. No significant correlations between the LDAA and aortic root dimension (*r* = .1, *p* = .409) and between the LDAA and diameter of the ascending aorta (*r* = .16, *p* = .170) were found in the examined patients (Fig. 4).

### Intra- and inter-observer and variability

The interobserver variability for the longitudinal stretching of the ascending aorta was 0.95 (95% CI: 0.92–0.97) and the intraobserver variability was 0.96 (95% CI: 0.94–0.98).

## Discussion

This is the first study to evaluate the longitudinal displacement of the aortic annulus and aortic root using magnetic resonance imaging in patients from all age groups. The longitudinal displacement of the aortic annulus is regarded as an important factor that has an impact on the wall stress in the ascending aorta [10, 12, 13]. The LDAA may be responsible for a transverse intimal entry tear in the ascending aorta that is a common finding in patients with type A aortic dissection [13].

The results of this study can be summarized as follows:

(i) the measurement of the LDAA using the magnetic resonance imaging is feasible,
(ii) human aortic root and ascending aorta are significantly stretched during systole,
(iii) the distance to which the aorta is stretched decreases with age,
(iv) the LDAA does not correlate with the dimensions of aortic root and ascending aorta.

The most common parameter used to evaluate the risk of aortic dissection is the maximum diameter of the vessel [1–3, 15, 16]. There is a correlation between the diameter of the aorta and the risk of dissection, however, most dissections occur in patients with only slightly enlarged aortas [1, 4, 5]. This phenomenon can be explained by the fact that there are potentially many more patients with moderately dilated aortas compared to those with large aneurysms. The risk is lower when the vessel is not very dilated but the overall number of dissections is still much higher in patients with moderately dilated aortas [4, 5]. Thus, new parameters to assess the risk of dissection are needed to better evaluate this risk in patients with moderately dilated aortas.

Human ascending aorta is subjected to many biomechanical factors. Some of them, including the geometry of the vessel, arterial blood pressure and longitudinal systolic stretching due to heart motion play the key role in the biomechanics of this vessel [13, 17–20].

The longitudinal displacement of the aortic annulus has only been evaluated using the aortography, which is an invasive procedure that requires heart catheterization and contrast injection [10, 11]. In our study, we proposed a method to assess this parameter using the CMR imaging in the coronal cine sequences. The measurements were easy to perform and are reproducible with satisfactory inter- and intraobserver variability.

The LDAA was on average 11.6 mm and did not differ significantly between males and females. The maximum dimension of the vessel did not affect the longitudinal displacement of the aortic annulus. In our study, the LDAA negatively correlated with age. It may be explained by the fact that aortic wall elasticity decreases with age [21]. The LDAA could be used as a parameter to predict the potential loss of elasticity of the ascending aorta, i.e. in young patients. An unusually low value of the LDAA may then indicate that the aorta has lost its normal elasticity and that such a patient should be examined more thoroughly in search for connective tissue disorders. The LDAA could be a reasonable substitute for standard arterial stiffness to examine the elasticity/stiffness of the aorta and not the stiffness of the whole arterial tree. Nevertheless, additional studies are necessary to confirm the correlation between aortic wall elasticity and LDAA.

### Study limitations

This is a small cohort study that include normal controls. An additional study assessing the LDAA in patients’ with cardiovascular diseases is necessary to evaluate the clinical relevance of the LDAA. Due to a small sample size, one cannot rule out that the significant correlation of the LDAA with age may be influenced by other confounding factors. A study on a much larger population is warranted. The measurements of the LDAA were performed on two-dimensional images. However, the aortic annulus is a three-dimensional structure that changes its position during the heart cycle. It might have influenced the accuracy of the measurements.

## Conclusions

In conclusion, the measurement of the longitudinal displacement of the aortic annulus using the CMR imaging is feasible and reproducible. Human aortic root and ascending aorta are significantly stretched during systole and the distance to which the aorta is stretched decreases with age. The LDAA does not correlate with the dimensions of aortic root and ascending aorta. In our opinion, the LDAA is a biomechanical factor that should be taken into account when analyzing the biomechanics of the thoracic aorta.

## Abbreviations

CMR: Cardiovascular magnetic resonance
ECG: Electrocardiogram
FIESTA: Fast Imaging Employing Steady-state Acquisition
FOV: Field of view
LDAA: Longitudinal displacement of the aortic annulus
STJ: Sino-tubular junction
VAJ: Ventriculo-aortic junction
